# Stepwise Optimization of the Procedure for Assessment of Circulating Progenitor Cells in Patients with Myocardial Infarction

**DOI:** 10.1371/journal.pone.0030389

**Published:** 2012-01-17

**Authors:** Yu-Xin Cui, Tom Johnson, Andreas Baumbach, Barnaby C. Reeves, Chris A. Rogers, Gianni D. Angelini, Debbie Marsden, Paolo Madeddu

**Affiliations:** Bristol Heart Institute, University of Bristol, Bristol Royal Infirmary, Bristol, United Kingdom; University of Padova, Medical School, Italy

## Abstract

**Background:**

The number and functional activity of circulating progenitor cells (CPCs) is altered in diabetic patients. Furthermore, reduced CPC count has been shown to independently predict cardiovascular events. Validation of CPCs as a biomarker for cardiovascular risk stratification requires rigorous methodology. Before a standard operation protocol (SOP) can be designed for such a trial, a variety of technical issues have to be addressed fundamentally, which include the appropriate type of red blood cell lysis buffer, FMO or isotype controls to identify rare cell populations from background noise, optimal antibody dilutions and conditions of sample storage. We herein propose improvements in critical steps of CPC isolation, antigenic characterization and determination of functional competence for final application in a prospective investigation of CPCs as a biomarker of outcome following acute myocardial infarction.

**Methods and Findings:**

In this validation study, we refined the standard operating procedure (SOP) for flow cytometry characterisation and functional analysis of CPCs from the first 18 patients of the *Progenitor Cell Response after Myocardial Infarction Study* (ProMIS). ProMIS aims to verify the prognostic value of CPCs in patients with either ST elevation or non-ST elevation myocardial infarction with or without diabetes mellitus, using cardiac magnetic resonance imaging (MRI) for assessment of ventricular function as a primary endpoint. Results indicate crucial steps for SOP implementation, namely timely cell isolation after sampling, use of appropriate lysis buffer to separate blood cell types and minimize the acquisition events during flow cytometry, adoption of proper fluorophore combination and antibody titration for multiple antigenic detection and introduction of counting beads for precise quantification of functional CPC activity in migration assay.

**Conclusion and Significance:**

With systematic specification of factors influencing the enumeration of CPC by flow cytometry, the abundance and migration capacity of CPCs can be correctly assessed. Adoption of validated SOP is essential for refined comparison of patients with different comorbidities in the analysis of risk stratification.

## Introduction

Myocardial infarction (MI) is the irreversible necrosis of cardiac muscle following prolonged ischemia. It remains one of the most common causes of morbidity and mortality despite new treatment and management systems [1]. Circulating biomarkers have been the focus of recent research in order to improve risk assessment, diagnosis, and prognosis of cardiovascular disease. Single or combined biomarkers have been also used for prediction of functional outcome after an MI, as verified by echocardiography or cardiac magnetic resonance imaging (MRI). However, circulating biomarkers suffer a number of limitations. The introduction of cellular biomarkers may represent an important advancement in the evaluation of risk stratification, because the number and function of progenitor cells could directly and simultaneously address pathogenic and reparative mechanisms [2].

In particular, circulating progenitor cells (CPCs) have been proposed to be associated with occurrence and prognosis of MI [3]. Subsets of this heterogeneous population possess pluripotent potential and may help myocardial healing by direct participation in cardiac neovascularisation and remodelling, whereas the monocyte component is seemingly engaged in the paracrine modulation of the above phenomena [4]. Strong evidence indicates that CPCs are mobilized after an MI in response to cytokine stimulation, with the extent of mobilization being positively correlated with indices of cardiac contractility during recovery [5], [6], [7]. Risk factors may decrease CPC mobilization after a heart attack, but the findings of studies investigating CPC mobilization in diabetes are controversial. A negative correlation between CPC number and cardiovascular complications was found in Type 2 diabetes [8], [9]. Other studies suggest that the viability and migration capacity of CPCs are impaired in diabetic patients, whereas CPC counts are not dramatically altered [10], [11], [12]. Furthermore, the usefulness of CPCs for cardiovascular risk stratification in diabetic versus non-diabetic patients remains undefined.

We have designed an observational clinical trial, the *Progenitor cell response after Myocardial Infarction Study* (ProMIS), to verify the prognostic value of CPCs in post-MI patients with or without type 2 diabetes, using cardiac MRI as primary endpoint. Here, we report the introduction of a refined SOP for optimization of CPC isolation, enumeration and qualification in functional assays

## Results

**Figure 1:**
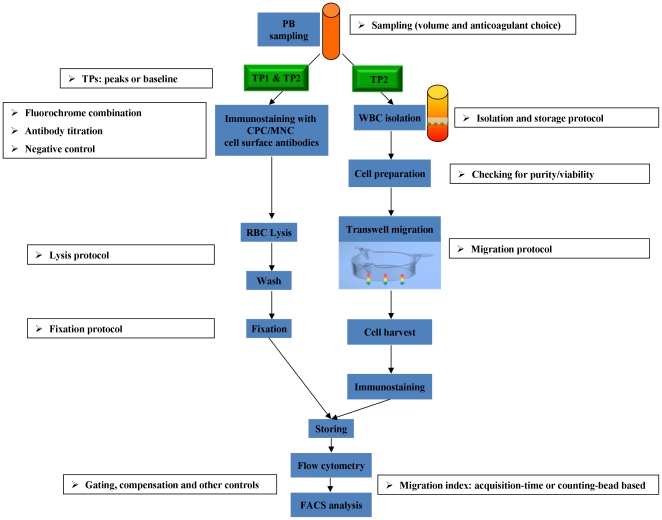
SOP of the ProMIS. Left: Characterization of circulating progenitor cells (CPCs) and monocytes in peripheral blood of MI patients. Right: Evaluation of migration capacity of CPCs by flow cytometry. PB, peripheral blood; TP, time point; WBC, white blood cell; CPC, circulating progenitor cell; MNC, monocyte; RBC, red blood cell.

Analyses were carried out on the first patients recruited to the ProMIS study, without knowledge of the diabetic status or MI classification (i.e. ST segment elevation myocardial infarction - STEMI - or Non-ST Segment Elevation Myocardial Infarction -NSTEMI -). In order to provide reliable and reproducible data for this trial, we first designed the experimental strategy based on the literature and our previous experience. Furthermore, to fulfil the requirements of the International Conference on Harmonisation (ICH) - Good Clinical Practice (GCP), we considered all the critical issues relevant to each individual step of the SOP as shown in **Figure 1**. Particularly, we validated or optimized some critical technical points that have potential effects on the ultimate outcome of the trial in a sequential manner.

### Difference in FACS layout of lyse/wash and lyse/no-wash procedures of blood samples

**Figure 2:**
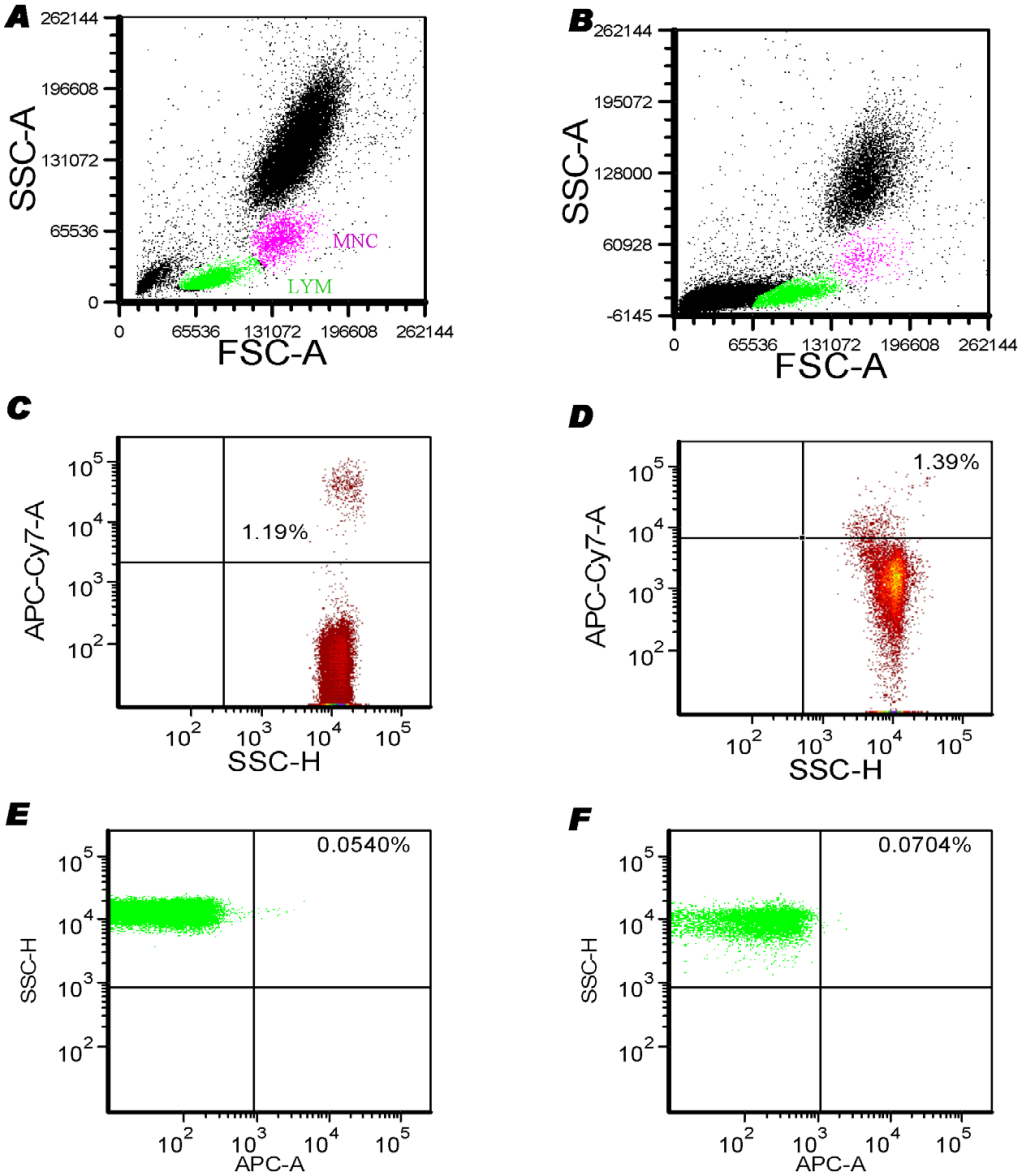
Comparison of lyse-wash and lyse-no-wash procedures. Whole blood samples were stained with progenitor cell markers and eFluor 780 fixable viability dye. Following RBC lysis with FCS lysis buffer (BD Bioscience), stained samples were washed with PBS (**A**, **C** and **E**) or stored at 4°C for subsequent FACS analysis within 5 days (**B**, **D** and **F**).

After immunostaining of human peripheral blood samples with cell surface markers, RBCs are often lysed in order to separate blood cell types and minimize the acquisition events during flow cytometry. We first investigated whether the simplified lyse/no-wash procedure, which reduces sample manipulation, would give improved cell yield over lyse/wash. As shown in **Figure 2**, from the samples subjected to lyse/wash step, lymphocyte and monocyte populations were separated nicely on the plot of FSC *vs.* SSC thus allowing easy gating of those cell sub-populations (**Figure 2A**). From the samples subjected to lyse/no-wash step, lymphocyte and monocyte populations could still be detected on the plot of FSC *vs.* SSC (**Figure 2B**); however, they were not as distinctive as the profile from the lyse/wash step. When dead cells were stained with the Fixable Viability Dye eFluor® 780, they could be better identified from the lyse/wash step (**Figure 2C**) than from the lyse/no-wash one (**Figure 2D**). However, the abundance of CD133^+^ cells enumerated by both methods was similar (**Figure 2E & F**). Furthermore, we found that the samples prepared by the lyse/no-wash approach frequently blocked the fluid system in the flow cytometer and it took much longer to acquire a given number of events.

### Comparison of RBC lysis buffers

**Figure 3:**
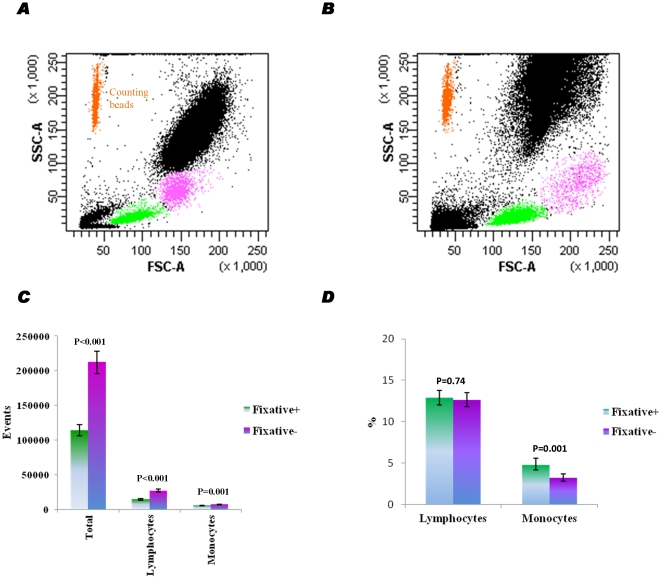
Effect fixative-containing RBC lysis buffer on cell yield. (**A**) Typical scatter plot of samples treated with fixative-containing lysis buffer. (**B**) Typical scatter plot of samples treated with fixative-free lysis buffer. (**C**) Effect of fixative in RBC lysis buffer on cell yield during FACS acquisition using counting beads compensation. (**D**) Effect of fixative in RBC lysis buffer on the percentage of lymphocyte and monocyte populations. Data are presented as geometric means with 95% confidence intervals.

There are two main types of RBC lysis buffers, i.e. with or without a fixative such as paraformaldehyde. We investigated the effect of fixative in the RBC lysis buffers on cell characterization by FACS in 3 replicates from each of 3 patients (18 samples in total; 3 patients×3 replicates×2 lysis buffers). As shown in **Figure 3**, typical blood cell populations were separated well using either a fixative-containing lysis buffer (BD) or a fixative-free variant (Invitrogen). However, the distributions of the typical cell populations were not equally distinctive in the scatter plots of FSC vs. SSC. It appeared that blood cells treated with fixative-free lysis buffer spread more along the scattergram (**Figure 3B**) as compared with the fixative-containing one (**Figure 3A**). After normalization by counting beads, samples treated with fixative-free lysis buffer yielded approximately 1.85-fold higher levels of total cells (P<0.001) and lymphocytes (P<0.001) and 1.28 fold higher levels of monocytes (P = 0.001) (**Figure 3C**). As shown in **Figure 3D**, the percentage of lymphocytes over total cells was similar between the two experimental conditions (P = 0.74), whereas the percentage of monocytes was lower in the samples using the fixative-free lysis buffer as compared with the fixative-containing lysis buffer (P = 0.001).

### Interference of the multiple antibodies conjugated with different fluorophores

**Figure 4:**
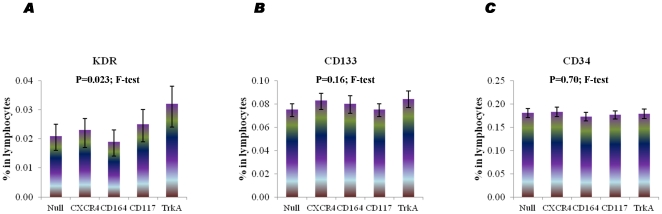
Representative graphs showing the interference of different fluorophore-conjugated antibodies. Different PE-labelled antibodies were added to KDR-FITC, CD133-APC and CD34-PECy7 in individual samples. Triplicate tests were performed. Data are presented as geometric means with 95% confidence intervals.

Because there is no single antigenic marker for CPC identification, multiple antibodies conjugated with a panel of fluorophores are commonly used for flow-cytometry profiling. Therefore, it is extremely important to test and eventually minimize the interference among different fluorophores. Particularly, we investigated whether different PE-conjugated antibodies (CXCR4, CD164, CD117 and TrkA) have an effect on the enumeration of three commonly-used progenitor cells markers, KDR-FITC, CD133-APC and CD34-PE-Cy7 in individual samples (60 samples in total; 4 patients×3 replicates×5 antibodies). As shown in **Figure 4A**, the presence of the selected PE conjugated antibodies appeared to have an effect on enumeration of KDR-FITC positive cells (P = 0.023; F-test). However, the presence of those PE-conjugated antibodies had no effect of the quantification of the selected cell populations expressing CD133-APC (P = 0.16; F-test) or CD34-PE-Cy7 (P = 0.70; F-test), as shown in **Figure 4C** and **Figure 4B**, respectively.

### Fluorescence-minus-one controls are necessary to quantify rare circulating progenitor cells

**Figure 5:**
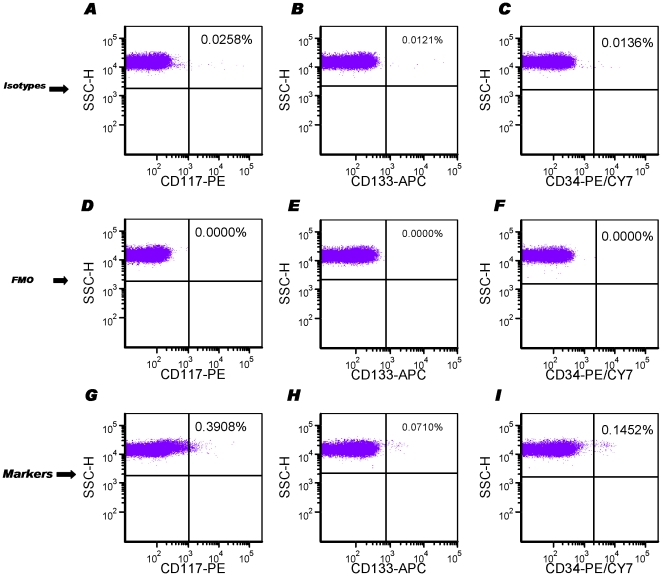
Fluorescence minus One (FMO) controls for the quantification of rare CPC events by FACS. Isotype controls were prepared by the combination of all the individual fluorophore-conjugated isotype control antibodies (**A**, **B** and **C**). FMO controls were prepared like isotype controls without adding a particular fluorophore-conjugated isotype control antibody, for instance fluorescence minus PE (**D**), fluorescence minus APC (**E**), fluorescence minus PE-Cy7 (**F**), respectively. The gating for each channel was defined to exclude nearly all background from the FMO controls on the 2D plots of FSC-H *vs.* individual fluorescence (e.g. PE, APC or PE-Cy7). Certain cell populations including CD117+ (**G**), CD133+ (**H**) and CD34+ (**I**) from a single sample were enumerated according to the gating strategy as above.

CPCs are a relatively rare component of the circulating leukocyte population even after mobilization following an acute MI. The fluorescence-associated rare events can be easily shadowed by auto-fluorescence or spillover contamination from other fluorophores. Therefore, when different fluorophore-conjugated antibodies are combined together, it is important to apply appropriate controls for background subtraction. We particularly investigated the effect of fluorescence minus one (FMO) controls and full isotype controls. As shown in **Figure 5 (A, B and C)**, the combinations of isotype controls showed background noise on each single channel involved (i.e. PE, APC and PE-Cy7), probably due to potential non-specific binding and/or fluorophore spillover contamination. Importantly, reference to the individual FMO controls allowed defining the appropriate gating (**Figure 5 D, E and F**), for determination of the number of a particular cell population (**Figure 5 G, H and I**). When tandem fluorophores such as PE-Cy7 were employed, isotype controls showed relatively higher background noise (**Figure 5C**), therefore the role of a FMO control was even more relevant (**Figure 5F**).

### Titration of each individual antibody is essential before combination for CPC enumeration

**Figure 6:**
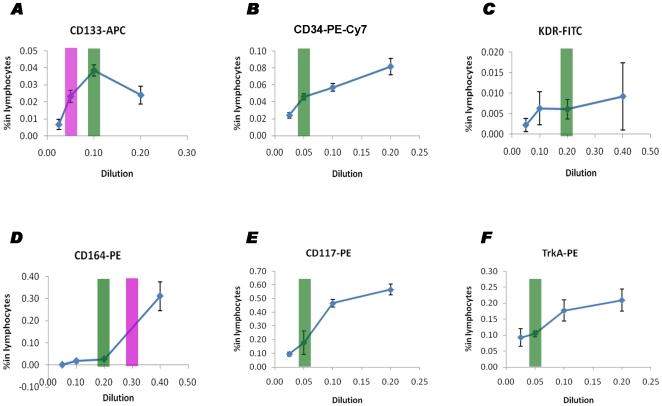
Titration of individual CPC markers before multicolour flow cytometry. Data are presented as geometric means with 95% confidence intervals. When without dilution, the volume of each antibody used for staining 100 µ0 of blood is: 10 µ0 of CD133-APC (50 CD133), 5 µL of CD34-PECy7 (50 µg/mL), 20 µL of KDR-FITC (50 µg/mL), 20 µL of CD164-PE (50 µg/mL), 5 µL of CD117-PE (50 µg/mL), and 20 µL of CD164-PE (25 µg/mL), respectively. The green bars show the dilution ratio of individual antibodies as recommended by vendors. The purple bars show the optimal dilution ratio of some antibodies according to our titration tests.

Before selected antibodies were applied for CPCs enumeration, we validated their dose dependence by serial titration. As shown in **Figure 6**, the signals obtained from some antigenic markers such as CD34, CD117 and TrkA are typically dose-dependent and reach saturation when a high dose of antibodies is used. The signal from samples stained with CD133 however reached a steep peak followed by gradual decline when antibody concentration was increased, which fits a parabolic-curve like pattern. The signal from samples stained with CD164 was blunted unless a high concentration of antibody was used. The signal from samples stained with KDR was always dim even when an excess of antibody was used. The optimal concentration of each individual antibody was not always as recommended by the vendor (Green Bar, **Figure 6**) thus implying the necessity of validation by the customer.

### The freshness and quality of the PB samples are relevant for the evaluation of CPCs

**Figure 7:**
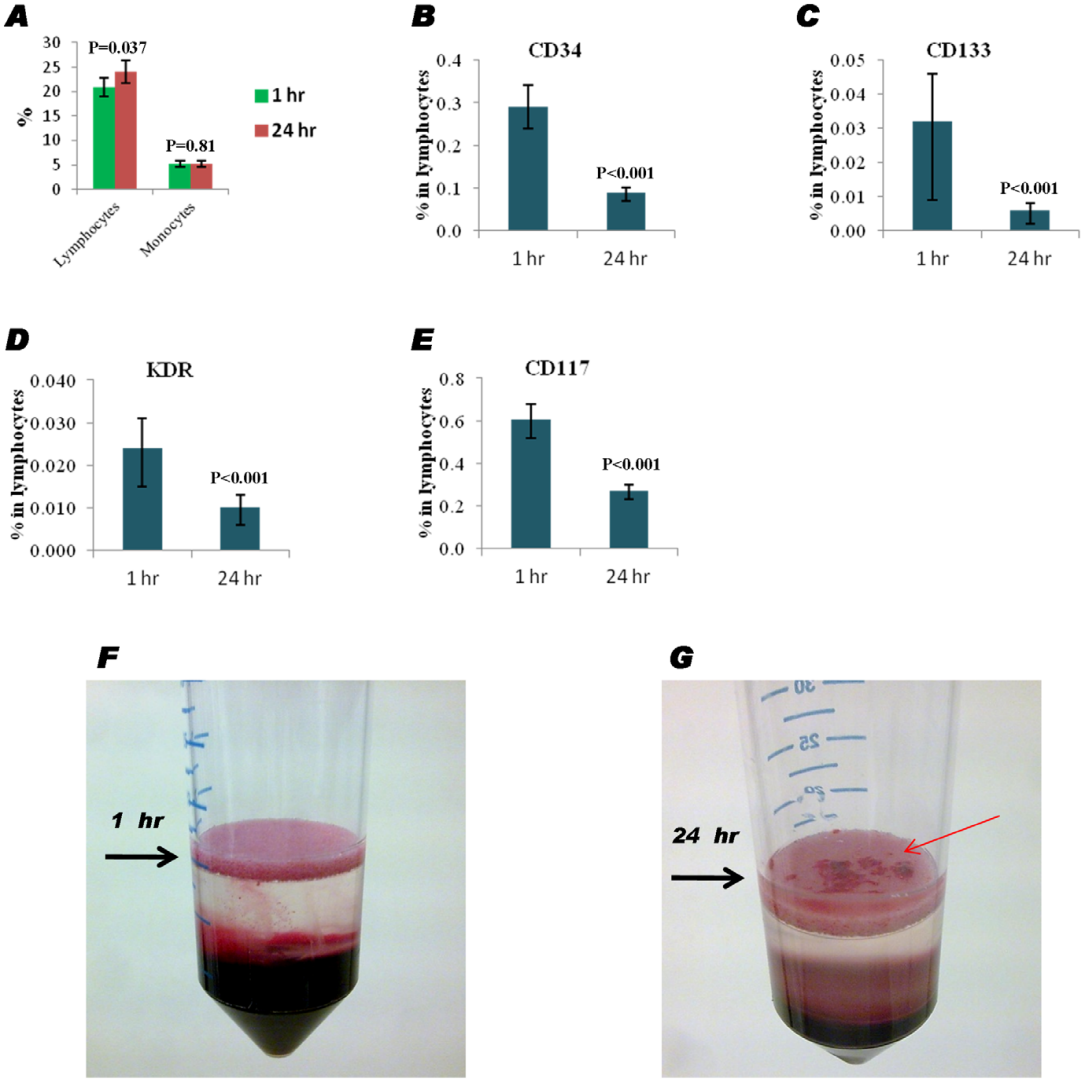
Effect of blood storage time on CPC profiling. Peripheral blood samples from MI patients were processed either directly (<1 hr) or stored at 4°C for 24 hr before analysis. (**A**) Bar graph showing the percentage of lymphocyte and monocyte cell populations. (**B**, **C**, **D** and **E**) Bar graph showing the percentage of CD34-PE-Cy7, CD133-APC, KDR-FITC and CD117-PE in lymphocytes, respectively before and after storage for 24 hrs. (**C** and **D**) Representative images show that there was excessive clotting in the Leucosep tubes after leukocyte isolation when samples were stored for 24 h at 4°C. Data are presented as geometric means with 95% confidence intervals.

Patients with MI refer to the clinic as emergencies at any time of day or night. Therefore, in order to maximise the opportunity for recruiting patients into a study like ProMIS, we investigated the effect of storing samples at 4°C for 24 hr before subjecting samples to immunostaining for flow cytometry analysis using data from 4 patients (24 samples in total; 4 patients×3 replicates×2 time points). As shown in **Figure 7**, it appeared that there is higher percentage of lymphocytes, but no change of the monocyte population after storage for 24 hrs (**Figure 7A**). Furthermore, the storage dramatically affected the quantification of distinct CPC populations with a CD34^+^, CD133^+^, KDR^+^ or CD117^+^ phenotype (**Figure 7B–E**). After mononuclear cells were isolated for functional tests such as migration assay, freshly processed samples appeared clear (**Figure 7F**), while clotting blocks were often observed from the tube containing a stored sample (**Figure 7G**). Clotting also occurred when venipuncture was technically difficult. These samples yielded artificially low levels of CPCs by flow cytometry.

### Evaluation of migration index using counting beads and acquisition time

**Figure 8:**
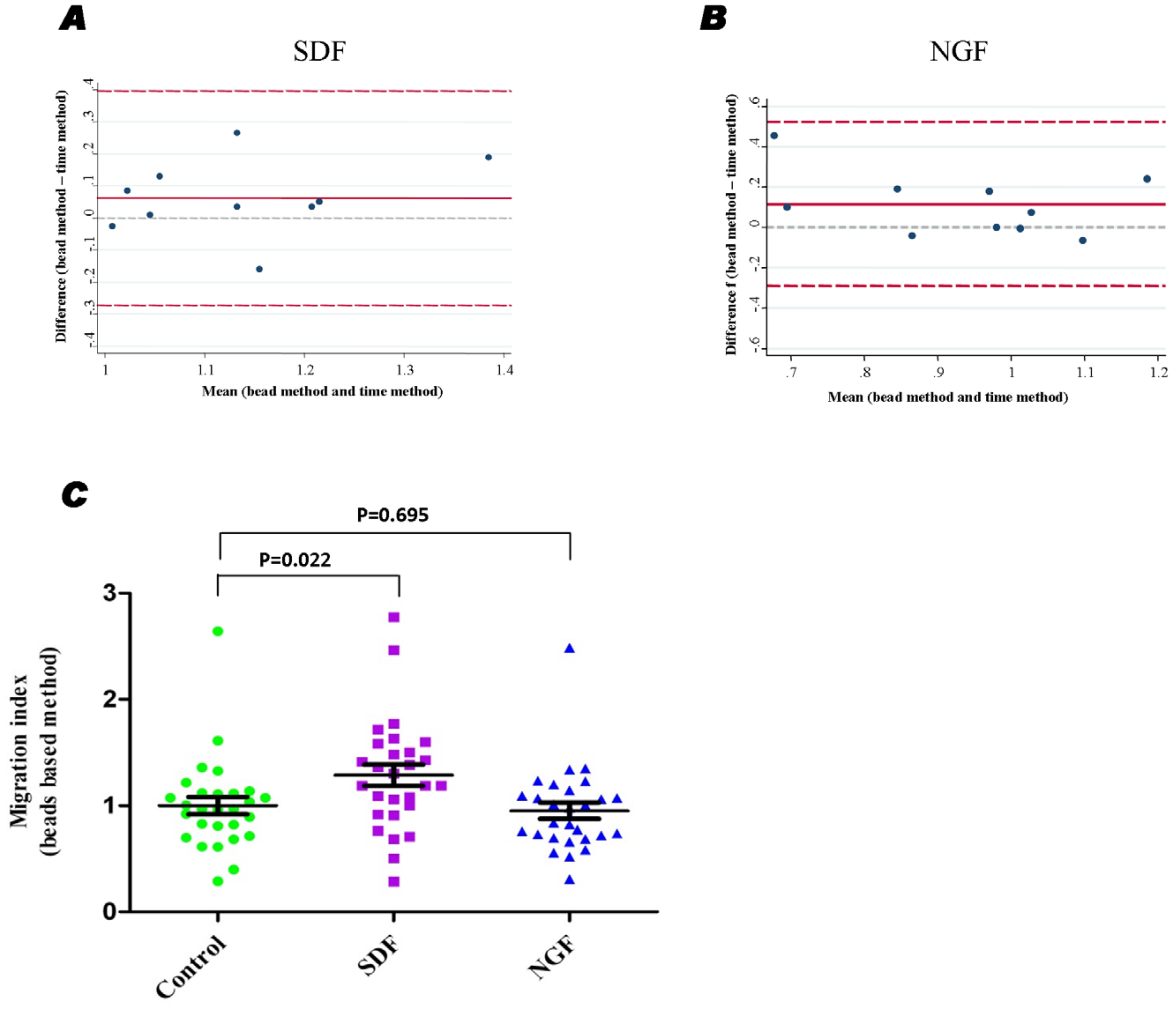
Migration capacity of isolated leukocytes assessed using time of acquisition by FACS and counting beads. Blood was collected at TP2 (84–108 h) post-MI and subjected to Histopaque-1077 density gradient centrifugation. The buffy coat was collected and washed in PBS. Cells were suspended in EBM-2 medium at a density of 0.5–1×10^7^ cells/mL and loaded on 6-well tissue culture inserts (3-µm pore ThinCert; Greiner Bio One). Medium with or without chemoattractants (*i.e.* SDF or NGF) was added in the bottom part of the inserts. Following transwell migration in a humidified CO_2_ incubator for 18 hrs, the migrated and non-migrated cells number was evaluated by FACS analysis with the assistance of acquisition time or counting beads. As described in **Materials and Methods**, migration index (ratio of migrated cell fraction in the total cells) of each sample was then estimated using both the acquisition time-based and beads-based approaches (**A** and **B**). Migration index evaluated in the presence of counting beads (**C**).

The migration assay is routinely used to estimate one fundamental functional property of mononuclear cells or isolated CPCs, e.g. the responsiveness to a chemoattractant. Flow cytometry can be subsequently applied to characterize distinct CPCs in the migrated and non-migrated fractions. However, it is not possible to quantify absolute cell numbers directly using the majority of flow cytometers such as a FACS Canto II. Therefore, following transwell migration of leukocytes toward SDF-1 or NGF, we compared the migration indexes which are normalized by using either the addition of counting beads or calculating acquisition time by the flow cytometer in 20 samples (10 patients×2 replicates). When SDF-1 was used as chemoattractant, the Bland-Altman plot was shown in **Figure 8A** with the 95% limits of agreement between two techniques ranging from −0.27 to +0.40. When NGF was used as a chemoattractant, the Bland-Altman plot was shown in Figure 8B with the 95% limits of agreement between the two techniques ranging from −0.30 to +0.52 (**Figure 8B**). These data indicate that both techniques can be used to evaluate migration activity of CPCs to SDF-1 or NGF-1. However, the acquisition method is based on the assumption of consistent speed during data collection, which is difficult for normalization because approximately just 11.59% of cells migrated to SDF (95% CI of geometric mean, 9.69–13.86%, n = 28), and the flow cytometric speed should be adjusted to allow detection accuracy for the non-migrated samples while reaching maximal acquisition for the migrated samples. Therefore, it may be more reliable to incorporate counting beads in order to precisely calculate the number of cells. Using the counting-bead based method, the migration of PB WBCs with response to SDF-1 or NGF was evaluated (**Figure 8C**). We demonstrated that the PB WBCs from MI patients migrated to SDF-1 but not NGF, which were indicated by one way ANOVA with LSD post hoc correction (P = 0.022 for SDF-1 and P = 0.695 for NGF, n = 28).

## Discussion

This study describes several technical implementations for enumerating and characterising CPCs in the ProMIS trial.

We first demonstrated that the lyse-wash step is superior to the lyse-no-wash step after immunostaining of peripheral blood. Using the lyse-and-wash technology, blood cell populations can be separated better and dead cells can be easily excluded either by a FSC-SSC plot or a fluorophores-conjugated viability dye. Furthermore, the sample solution prepared by a lyse-no-wash step is sticky and can block the fluid system of a cytometer like the BD Canto II. Samples need to be highly diluted with H_2_O when a lyse-no-wash method is applied, which results in extension of the running and analyse time of flow cytometry and thus becomes cost-inefficient. However, we acknowledge the appropriateness of the lyse-no-wash technique under specific circumstances; it is clear that the minimal manipulation of blood cells leads to less cell loss and less disturbance of fragile cells, which is particularly the case when one or two-colour markers (e.g. CD34) are of interest and it is not necessary obtain a large number of captured cell events (e.g. > = 1×10^5^ lymphocytes) [14], [15]. However, when multicolour antigenic analysis is required and time/cost effects have to be considered, the lyse-wash technique may provide more advantages [16].

Next, we compared the effect of RBC lysis buffers with or without the fixative paraformaldehyde. The fixative-free RBC lysis buffer markedly increased the yield of the total cells and lymphocyte population. The presence of paraformaldehyde in the lysis buffer did not have an effect on the yield of monocytes by flow cytometry. Fixation during RBC lysis leaves white cell populations (e.g. CD34^+^ cells) in sticky blood and leads to their loss during washing and resuspending, which might explain why the fixative-free lysis buffer provides a higher yield of cells after the lyse-wash procedure [17]. Therefore, fixative-free RBC lysis buffer is definitely the best option for the quantification of rare progenitor cells in blood by flow cytometry.

Although flow cytometry equipped with multiple lasers and fluorescence detectors is sensitive enough to identify rare cells in blood, variation in data characterising CPCs can be caused by various factors such as autofluorescence, cell aggregation, non-specific immunostaining of other cell types including dead cells/debris, non-lysed erythrocytes, platelets as well as system fluctuation due to excessive speed, inadequate cleaning or blocking. We tested the reproducibility of a cell population indicated by a fluorophores-conjugated marker in the presence of other markers by repetition. Sometimes other fluorophore-conjugated antibodies can interfere with quantification of CPCs. Therefore, when a study is carried out, protocols must be designed with strict validation of antibodies, which must be used consistently. According to good laboratory practice, repetition (e.g. duplicate/triplicate tests) is ideal, yet flow cytometric data are often presented as a single test to reduce costs.

When rare CPC populations are enumerated, attention must be given to potential contamination during the sample process, for instance, immunostaining with multiple fluorophores. The technology of FMO controls has been reported to be necessary to accurately identify positive cells in the fully stained sample after multicolour flow cytometry [18], [19]. In a study on rare CPC enumeration, this is definitely the case. Without the FMO controls, it is also difficult to set up proper gating due to the accumulation of background noise by spectral spillover from irrelevant fluorophores. This is more obvious when tandem fluorophores such as PE-Cy7 are combined.

Due to the complexity inherent to CPC heterogeneity, the application of multiple antibodies is often necessary. However, the selection of different photospheres can still be tricky. Our titration data suggest that the efficiency of immunostaining using an individual rare progenitor cell marker can be highly heterogeneous. Before antibodies are selected and combined, it is essential to perform individual dose-titration tests in order to identify the optimal strategy.

Although fresh samples are ideal, researchers would like to understand whether CPCs in peripheral blood are stable over short periods of time since being able to store samples for a few hours makes a study more practicable. Therefore, we investigated the effect of blood freshness on CPC-marker expression. After 24 hours of storage at 4°C, both the percentage of lymphocytes and expression of most CPC markers (e.g. KDR, CD133 and CD117) decreased, suggesting that CPCs in peripheral blood are fragile or the antigen can be downregulated or internalized during storage. Furthermore, when white blood cells in 24-hour blood are isolated by Histopaque 1077, clotting can be observed on the porous barrier in a LeucoSep tube, which may potentially cause degradation of viable cells and deterioration of cellular functions in vitro. Therefore, when PB samples are used for the evaluation of CPCs, the gold standard should remain fresh blood, and ‘the fresher the better’ [20]. Practically, a same-working-day strategy (e.g. 8 hrs) should be applied for a study, but this operative window may remarkably reduce the possibility to recruit patients with an acute MI who can present at any time of day.

Application of flow-cytometry to the CPC migration assay provides the means to understand how many and which CPCs respond to a particular cytokine. However, reproducibility could be reduced due to cell loss during multiple and tedious manipulation during preparation of samples for analysis. In order to enable robust high-throughput studies and track migration in a real-time manner, some improved techniques emerged which include Oris™ Cell Migration Assay (Platypus technologies, Madison, WI) and Fluoroblok™ (BD Biosciences, Bedford, MA). These techniques would be encouraged to integrate cutting-edge high throughput migration methods with flow cytometry in future.

In conclusion, although the interest in CPCs is steadily increasing, the lack of a consensus on standardised protocols remains a barrier to clinical exploitation [21], [22]. In this study, we addressed some critical steps instrumental to optimal enumeration and characterisation of CPCs in cardiovascular patients. Our findings may be also applicable for other clinical trials when CPCs need to be enumerated, for instance, stroke, and chemo-/radio- therapies for cancer patients.

## Materials and Methods

### Study design

We selected four cohorts for assessment in ProMIS, namely patients presenting with either ST elevation MI (STEMI) or non-ST-elevation MI (NSTEMI), with or without type 2 diabetes mellitus. We are recruiting 18 patients prospectively for each cohort, thus 72 patients in total. Eligibility criteria are as follows: patients presenting with clinical features of STEMI or NSTEMI within 24 hours of symptom onset, aged 40 to 75 yrs on admission, and resident within 40 miles of the study centre. Patients are excluded if they have anaemia (haemoglobin<10 mg/dL), cardiogenic shock, significant renal impairment (eGFR<50 mL /min per 1.73 m^2^), haemodynamic instability, contraindications to undergoing a magnetic resonance scan (e.g. metallic implant, pacemakers, screws, claustrophobia *etc*), have had previous coronary event within the last 12 weeks, are participating in another clinical trial or show heightened anxiety during recruitment.

Written informed consent is obtained directly from each eligible patient before the trial. 10 mL blood sample (TP1 sample) is then obtained within 12–24 hours of the onset of symptoms for FACS analysis. A second 50 mL blood sample (TP2 sample) is obtained at 84–108 hours after the onset of symptoms for FACS analysis and transwell migration assay of CPCs. Blood samples are transferred to the laboratory at 4°C and processed within 24 hours unless described differently. Demographic data and clinical data including past medical history, medication history, acute investigations and treatment information are recorded in a case report form (CRF). Patients have a cardiac magnetic resonance (CMR) assessment on day 5 after presentation to determine the extent of MI injury (scar volume) and then 3 months later to assess the final volume of the scar. The protocol for this study was approved by the UK NHS Research Ethics Committee (REC reference number: 09/H0104/58).

### FACS analysis

One hundred µL of peripheral blood samples collected in ethylenediaminetetraacetic acid (EDTA) containing tubes are incubated for 30 min at 4°C with mouse-anti-human monoclonal antibodies including kinase insert domain receptor (KDR) conjugated with fluorescein isothiocyanate (FITC) (R&D Systems, Minneapolis, MN, USA), CD133/2 (clone 293C3) conjugated with allophycocyanin (APC) (Miltenyi Biotec, Bergisch Gladbach, Germany), CD34 (clone 8G12) conjugated with phycoerythrin-Cy7 (PE-Cy7) (BD Bioscience, San Jose, CA), CXCR4 (CD184) conjugated with PE (Invitrogen, Paisley, *UK*), CD164 (clone N6B6) conjugated with PE (Becton Dickinson, San Jose, CA), CD117/C-KIT (clone 104D2) conjugated with PE (Invitrogen, Paisley, UK), TrkA (Clone 165131) conjugated with PE (R&D Systems, Minneapolis, MN, USA), CD14 conjugated with FITC and CD16 conjugated with PE (Invitrogen, Paisley, UK) , respectively. Additional samples are stained with appropriate isotype control antibodies following the fluorescence-minus-one method. The isotype control antibodies we used were Mouse IgG1-FITC (Invitrogen, Paisley, UK), Mouse IgG1-PE (Invitrogen, Paisley, UK), Mouse IgG2b-APC (Miltenyi Biotec, Bergisch Gladbach, Germany) and Mouse IgG1 PE-Cy7 (BD Bioscience, San Jose, CA).

The following steps are then carried out in sequence:

- Cells are lysed with FACS lysis buffer (BD Bioscience, San Jose, CA) for the lyse/no wash procedure.
- For the lyse/wash procedure, samples are subjected to lysis of red blood cells (RBC) for 15 min at room temperature using either 1× FACS lysis buffer (BD Bioscience, San Jose, CA) or high yield fixative free lysis buffer (Invitrogen, Paisley, UK).
- Cells are spun down for 10 min at 300×*g* and then stained with the Fixable Viability Dye eFluor® 780 (eBioscience, San Diego, CA) for 30 min at 4°C.
- Cells are washed with PBS and fixed with 1% paraformaldehyde (PFA). AccuCheck counting beads are occasionally included for cell quantification according to the manufacturer's instructions (Invitrogen, Paisley, UK).
- Flow cytometric acquisition is performed on a FACS Canto II system (BD Bioscience, San Jose, CA) within one week. FACS analysis is carried out using the FACS Diva 6.0 software (BD Bioscience, San Jose, CA).

### Migration assay

Peripheral blood samples at TP2 in EDTA tubes are diluted with PBS supplemented with 2 mM EDTA and 0.5% BSA (v/v, 1∶1); 30 mL of diluted blood solution is then loaded in a 50-mL LeucoSep tubes (Greiner, Frickenhausen, Germany) with 15 mL of Histopaque 1077 (Sigma, Poole, UK) and centrifuged at 850×*g* for 25 min. The buffy coat layers containing white blood cells are collected and washed twice with sterile PBS. Cells are then resuspended in EBM-2 (Lonza, Berkshire, UK) supplemented with 0.1% BSA. 0.5–1×10^7^ cells/mL of cells are loaded onto the upper part of ThinCert™ tissue culture polystyrene inserts (452.4 mm^2^ culture surface, 3.0 µm pore size; Greiner BioOne, Gloucester, UK) which are pre-assembled on 6-well plates containing medium as above with or without 100 ng/mL of recombinant human SDF-1α or β-NGF (PeproTech, Rocky Hill, NJ), respectively. Cells are then incubated at 37°C in a humidified incubator with 5% CO_2_ for 18 hrs. Cells from both parts of the transwell inserts are dissociated with Accutase (Invitrogen, Paisley, UK) and collected into FACS tubes. Following washing using PBS, migrated and non-migrated cells are stained with KDR-FITC, CD133/2 (293C3)-APC, CD34 (8G12)-PE-Cy7 and CXCR4 (CD184)-PE antibodies for flow cytometry analysis as described above.

### Data analysis and statistics

Data are shown as geometric mean with 95% confidence intervals. For the evaluation of migration capacity, following normalization of the data using FACS requisition time or counting beads, migrated cells (in the down part of the inserts) were divided by the sum of the migrated cells and non-migrated cells (that were left in the upper part of the inserts) and multiplied by 100 to give the migration percentage. Migration index is the value of migration percentage in a sample divided by that in the control, *i.e.*,
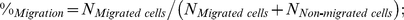


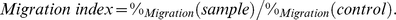
Statistical analyses were performed using regression modelling, taking account of the repeated measurements from the same patient. If the overall test for a difference between groups (F-test) was statistically significant (p<0.05) then paired comparisons between groups were made. Skewed data were transformed to the logarithmic scale for analysis and results are presented as geometric means with 95% confidence intervals. Model assumptions were checked and influential outlying values were excluded. Bland and Altman plots [13] were used to determine concordance between techniques of migration flow cytometric measurement with the limits of agreement defined as the mean of the difference ±1.96 standard deviations. It was considered statistically significant when P value<0.05. Analyses were carried out using Stata 11.2 (StataCorp LP 4905 Lakeway Drive College Station, Texas. USA).
